# Effect of 48‐week pemafibrate on non‐alcoholic fatty liver disease with hypertriglyceridemia, as evaluated by the FibroScan‐aspartate aminotransferase score

**DOI:** 10.1002/jgh3.12650

**Published:** 2021-08-28

**Authors:** Takeshi Hatanaka, Takashi Kosone, Naoto Saito, Satoshi Takakusagi, Hiroki Tojima, Atsushi Naganuma, Hitoshi Takagi, Toshio Uraoka, Satoru Kakizaki

**Affiliations:** ^1^ Department of Gastroenterology Gunma Saiseikai Maebashi Hospital Maebashi Japan; ^2^ Department of Gastroenterology and Hepatology Kusunoki Hospital Fujioka Japan; ^3^ Department of Gastroenterology and Hepatology Gunma University Graduate School of Medicine Maebashi Japan; ^4^ Department of Gastroenterology National Hospital Organization Takasaki General Medical Center Takasaki Japan; ^5^ Department of Clinical Research National Hospital Organization Takasaki General Medical Center Takasaki Japan

**Keywords:** albumin‐bilirubin score, FibroScan‐aspartate aminotransferase score, hypertriglyceridemia, non‐alcoholic fatty liver disease, pemafibrate

## Abstract

**Background and Aim:**

This retrospective study investigated the effect of 48‐week pemafibrate therapy in non‐alcoholic fatty liver disease (NAFLD) with hypertriglyceridemia, as evaluated by the FibroScan‐aspartate aminotransferase (FAST) score.

**Methods:**

A total of 31 NAFLD patients who were treated with pemafibrate in Gunma Saiseikai Maebashi Hospital and Kusunoki Hospital from September 2018 to April 2020 were included in the current study. We used the FAST score, which is a novel index of steatohepatitis that can be calculated based on the AST value, controlled attenuation parameter (CAP), and liver stiffness measurement (LSM), to evaluate the effect of pemafibrate treatment.

**Results:**

The median age was 64.0 (interquartile range [IQR] 55.0–75.0) years and 14 patients (45.2%) were male. Median body mass index was 26.8 (IQR 23.8–28.8). Hypertension and diabetes mellitus were detected in 14 (45.2%) and five (16.1%) patients, respectively. Fasting triglyceride and high‐density lipoprotein cholesterol were significantly improved (*P* < 0.001 and 0.013, respectively) and the AST, alanine aminotransferase (ALT), alkaline phosphatase, and γ‐glutamyl transpeptidase values were significantly decreased during pemafibrate treatment (*P* = 0.041, <0.001, <0.001, and <0.001, respectively). While the LSM value and CAP value did not differ to a statistically significant extent (*P* = 0.19 and 0.140, respectively), the FAST score was significantly improved during pemafibrate treatment (*P* = 0.029). The delta FAST score was found to be correlated with the variations of ALT (r = 0.504, *P* = 0.005), which represents the effect of pemafibrate.

**Conclusions:**

Pemafibrate improved the FAST score due to the hepatic anti‐inflammatory effect, indicating that pemafibrate may prevent disease progression in NAFLD patients with hypertriglyceridemia.

## Graphical abstract

The FibroScan‐aspartate aminotransferase (FAST) score, which stratifies patients with risk of progressive non‐alcoholic steatohepatitis effectively, was significantly improved during pemafibrate treatment. The delta FAST score was correlated with the variations of alanine aminotransferase, which represents the effect of pemafibrate. Pemafibrate may prevent disease progression in non‐alcoholic fatty liver disease patients with hypertriglyceridemia.

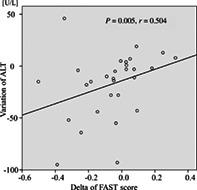

## Introduction

Non‐alcoholic fatty liver disease (NAFLD) is a common cause of chronic liver disease^1^; its prevalence is estimated to be 25% worldwide according to a meta‐analysis of studies reported from 2006 to 2014.^2^ Obesity, diabetes mellitus (DM), hyperlipidemia, and metabolic syndrome are well‐known risk factors for NAFLD.^3^ Non‐alcoholic steatohepatitis (NASH), which is defined as histological findings of ≥5% hepatic steatosis and inflammation with hepatocyte injury, increases the risk of developing cirrhosis, liver failure, and carcinogenesis.^3^ To date, the proportion of liver transplantation procedures performed for NASH patients is increasing in Western countries^4^, ^5^ and the prevalence of NAFLD and NASH are projected to increase globally with the continued high rate of obesity and DM.^6^ While bodyweight loss is the mainstream treatment for NASH, the response to lifestyle intervention is limited in clinical settings. In addition, a few effective pharmacotherapies for NASH have been established.^3^, ^7^

Pemafibrate, a novel selective peroxisome proliferator‐activated receptor alpha modulator (SPPARMα),^8^ has the effect of decreasing the concentration of triglyceride (TG) and increasing the concentration of high‐density lipoprotein cholesterol (HDL‐C).^9^, ^10^ Pemafibrate was also reported to significantly reduce alanine aminotransferase (ALT) and γ‐glutamyl transpeptidase (γ‐GTP).^11^ Recent studies have reported the efficacy and safety of pemafibrate for patients with NAFLD.^12^, ^13^, ^14^ We also revealed the efficacy and safety of pemafibrate for biopsy‐proven NASH patients.^15^ Although the study population was small and the duration of treatment was short in these previous studies, pemafibrate is expected to be a promising treatment for NAFLD patients with hypertriglyceridemia.

The FibroScan‐aspartate aminotransferase (FAST) score was reported to be a useful method for non‐invasively identifying NASH patients with significant activity (NAFLD activity score [NAS] ≥ 4) and advanced fibrosis (≥F2), who could benefit from pharmacotherapy.^16^ The FAST score is calculated based on the aspartate aminotransferase (AST) value, liver stiffness measurement (LSM), and controlled attenuation parameter (CAP) that stratifies patients with risk of progressive NASH effectively.^16^ However, the role of FAST score in NAFLD patients treated with pemafibrate has not been clearly elucidated. We expanded the patient cohort and the treatment period to investigate the efficacy of pemafibrate for NAFLD patients with hypertriglyceridemia who were evaluated by the FAST score.

## Methods

### Participated patients

The present retrospective study included a total of 31 NAFLD patients who were treated with pemafibrate in Gunma Saiseikai Maebashi Hospital (Maebashi, Gunma, Japan) and Kusunoki Hospital (Fujioka, Gunma, Japan) from September 2018 to April 2020. The following patients were included: patients diagnosed with fatty liver and hypertriglyceridemia; patients who did not report the presence or history of significant habitual alcohol intake (≥30 g/day for men and ≥20 g/day for women); their liver function was well‐preserved (not Child‐Pugh class B or C); and patients who were evaluated by the FAST score at pretreatment. We confirmed that all participated patients did not have gallbladder stones, evidence of hepatocellular carcinoma (HCC), and renal impairment before pemafibrate treatment. Among these 31 NAFLD patients, nine NASH patients who participated in our previous report^15^ were included in the present study.

The diagnosis of fatty liver was made based on the findings of abdominal ultrasonography (US), which included increased hepatic echogenicity, liver–kidney contrast, and deep US attenuation in the liver. Hypertriglyceridemia was also diagnosed based on an elevated blood concentration of fasting TG (≥150 mg/dL) or non‐fasting TG (≥175 mg/dL).^17^ The institutional review board of Gunma Saiseikai Maebashi Hospital and Kusunoki Hospital approved this retrospective study and waived the requirement for informed consent from the participants.

### Pemafibrate treatment

Patients were prescribed pemafibrate (oral, 0.1 mg, twice a day) and visited the outpatient clinic every 2–8 weeks. The patients also received a biochemical examination to investigate the lipid profile, liver function, and renal function every 1–2 months. We carried out transient elastography (FibroScan; ECHOSENS, Paris, France) to measure the LSM and CAP at pretreatment, at 12 weeks, at 24 weeks, and at 48 weeks. The Common Terminology Criteria for Adverse Events version 5.0 was used to evaluate adverse events (AEs) associated with pemafibrate.

### Calculation of the FAST score, ALBI score, and other parameters

The FAST score^16^ consists of the AST, LSM, and CAP and was calculated as e^x^/(1 + e^x^), where X = −1.65 + 1.07 × ln (LSM) + 2.66*10^−8^ × CAP^3^–63.3 × AST^−1^. Albumin‐bilirubin (ALBI) score^18^ was also calculated as the following formula; ALBI score = (log_10_ bilirubin [μmol/L] × 0.66) + (albumin [g/L] × −0.085). FIB‐4^19^, ^20^ and NAFLD fibrosis score (NFS)^21^ were also calculated according to the previous studies.

### Statistical analyses

Continuous data were expressed as the median (interquartile range) and categorical data were expressed as the number (percentage). Friedman test was used to analyze the multiple comparisons. When a significant extent was differed, post‐hoc analysis was conducted using Bonferroni method. Variation of parameters was calculated as the value at 12 weeks, 24 weeks, or 48 weeks—the value at pretreatment. The amount of variation was expressed as mean (95% confidence interval [CI]). The relationship between the variation of parameters was assessed by spearman's rank correlation coefficient. *P* values of <0.05 were considered to indicate statistical significance. All statistical analyses were performed using the IBM Statistical Package for Social Sciences software program (version 24, IBM SPSS 24, IBM, NY, USA).

## Results

### Patient characteristics

The median age was 64.0 (55.0–75.0) years and 14 patients (45.2%) were male. The median body mass index (BMI) was 26.8 (23.8–28.8) and 20 patients (64.5%) had a BMI of >25 (kg/m^2^). Hypertension and DM were detected in 14 (45.2%) and five (16.1%) patients, respectively. All DM patients received sodium‐glucose co‐transporter‐2 (SGLT2) inhibitor concomitantly. Vitamin E, renin‐angiotensin system (RAS) inhibitor, and statin were also used in four (12.9%), six (19.4%), and 11 patients (35.5%), respectively. Three patients (9.7%) switched from bezafibrate to pemafibrate and one patient (3.2%) switched from eicosapentaenoic acid and docosahexaenoic acid preparation to pemafibrate (Table 1). Ten patients (32.2%) received the percutaneous liver biopsy before pemafibrate treatment, resulting in a diagnosis of NASH.

**Table 1:** Patient characteristics

Variables	All patients (*n* = 31)
Age (years)	64.0 (55.0–75.0)
Males, *n* (%)	14 (45.2)
BMI (kg/m^2^)	26.8 (23.8–28.8)
BMI (kg/m^2^) > 25, *n* (%)	20 (64.5)
Metabolic diseases, *n* (%)	
Hypertension	14 (45.2)
Diabetes mellitus	5 (16.1)
Concomitant drugs, *n* (%)	
SGLT2 inhibitor	5 (16.1)
Thiazolidinedione	0 (0.0)
GLP‐1 agonist	0 (0.0)
Vitamin E	4 (12.9)
RAS inhibitor	6 (19.4)
Statin	11 (35.5)
Switch from the other antihyperlipidemic drug, *n* (%)	
Bezafibrate	3 (9.7)
EPA and DHA preparation	1 (3.2)

Data are expressed as the median (IQR).

BMI, body mass index; DHA, docosahexaenoic acid; EPA, eicosapentaenoic acid; GLP‐1, glucagon‐like peptide‐1; IQR, interquartile range; RAS, renin‐angiotensin system; SGLT2, sodium‐glucose co‐transporter‐2.

### Comparison between variables at pretreatment, 12 weeks, 24 weeks, and 48 weeks

The bodyweight and BMI were not significantly reduced during the pemafibrate treatment in all patients (*P* = 0.23 and 0.23, respectively). The values of AST, ALT, alkaline phosphatase (ALP) and γ‐GTP were significantly improved (*P* = 0.041, <0.001, <0.001, and < 0.001, respectively). While the significant extent was not found in total bilirubin, the serum albumin was significantly increased (*P* < 0.001), which resulted in a statistically significant improvement of the ALBI score (*P* < 0.001). With respect to the lipid profile, fasting TG and HDL‐C were significantly improved (*P* < 0.001 and 0.013, respectively) while low‐density lipoprotein cholesterol did not (*P* = 0.23). Regarding liver fibrosis markers, FIB‐4 and NFS were not significantly differed during the treatment. LSM numerically decreased during the pemafibrate treatment (*P* = 0.19). FAST score was significantly improved with a statistical significance (*P* = 0.029; Table 2).

**Table 2:** Comparison of values at pretreatment, 12 weeks, 24 weeks, and 48 weeks

	All patients (*n* = 31)					Patients who did not receive SGLT2 inhibitor (*n* = 26)				
Variables	Pretreatment	12 weeks	24 weeks	48 weeks	*P*	Pretreatment	12 weeks	24 weeks	48 weeks	*P*
Bodyweight	70.8 (59.7–76.7)	69.3 (59.0–78.6)	68.9 (57.2–79.4)	70.9 (58.5–81.8)	0.23	67.7 (58.2–75.2)	68.7 (57.5–75.5)	66.1 (56.2–74.3)	69.0 (57.4–77.3)	0.17
BMI	26.8 (23.8–28.8)	26.3 (23.8–28.4)	26.0 (23.9–28.2)	26.3 (23.8–28.3)	0.23	25.8 (23.2–27.8)	26.0 (23.4–27.6)	25.6 (23.5–27.5)	26.0 (23.6–28.2)	0.18
AST (U/L)	41 (24–53)	35 (28–47)	30 (24–41)	34 (24–48)	0.041	42 (24–56)	35 (27–48)	32 (24–41)	38 (26–49)	0.107
ALT (U/L)	49 (25–66)	33 (23–52)	25 (18–47)	32 (18–49)	<0.001	50 (25–70)	33 (23–53)	25 (18–47)	33 (21–49)	<0.001
ALP (U/L)	242 (181–296)	179 (135–250)	172 (128–203)	151 (126–196)	<0.001	246 (181–303)	176 (134–224)	177 (127–206)	153 (126–211)	<0.001
γ‐GTP (U/L)	55 (32–104)	32 (18–56)	32 (21–47)	31 (21–43)	<0.001	56 (34–103)	31 (20–49)	29 (23–43)	30 (22–41)	<0.001
Total bilirubin (mg/dL)	0.73 (0.60–0.90)	0.67 (0.60–0.79)	0.69 (0.53–0.90)	0.70 (0.60–0.80)	0.064	0.73 (0.58–0.86)	0.66 (0.59–0.76)	0.68 (0.58–0.91)	0.70 (0.56–0.80)	0.25
Albumin (g/dL)	4.4 (4.1–4.6)	4.5 (4.2–4.7)	4.5 (4.2–4.7)	4.6 (4.3–4.7)	<0.001	4.4 (4.1–4.6)	4.5 (4.2–4.7)	4.6 (4.2–4.8)	4.6 (4.2–4.7)	<0.001
ALBI score	−2.96 (−3.20 to −2.69)	−3.05 (−3.28 to −2.86)	−3.10 (−3.33 to −2.90)	−3.15 (−3.30 to −2.91)	<0.001	−3.00 (−3.20 to −2.69)	−3.08 (−3.30 to −2.85)	−3.14 (−3.34 to −2.90)	−3.14 (−3.29 to −2.93)	<0.001
Fasting TG (mg/dL)	172 (153–227)	108 (83–135)	88 (80–110)	99 (81–129)	<0.001	168 (147–230)	100 (81–121)	88 (80–115)	93 (71–128)	<0.001
LDL‐C (mg/dL)	114 (88–134)	104 (88–120)	103 (90–122)	102 (88–114)	0.23	112 (95–141)	105 (92–121)	104 (91–123)	106 (87–114)	0.120
HDL‐C (mg/dL)	50 (43–63)	57 (47–62)	58 (47–72)	61 (45–68)	0.013	52 (43–63)	57 (47–62)	59 (46–73)	61 (45–69)	0.025
Creatinine	0.80 (0.62–0.94)	0.80 (0.63–0.93)	0.81 (0.66–0.94)	0.83 (0.68–0.93)	0.72	0.80 (0.61–0.95)	0.79 (0.61–0.94)	0.81 (0.62–0.93)	0.79 (0.61–0.93)	0.84
eGFR	68.3 (57.9–82.4)	65.9 (59.1–84.0)	66.3 (56.2–82.5)	66.3 (57.0–80.3)	0.42	71.0 (57.8–83.6)	66.6 (57.7–84.9)	67.6 (57.3–83.0)	68.4 (58.7–81.0)	0.61
Platelet count (×10^4^/μL)	21.5 (15.6–26.3)^†^	23.1 (18.3–27.0)	22.7 (19.2–29.2)	22.4 (18.8–25.6)	0.001	21.5 (17.2–25.8)	23.1 (19.8–27.0)	23.1 (20.2–29.5)	22.4 (19.3–25.6)	<0.001
FIB‐4	1.62 (1.03–2.95)^†^	1.70 (0.93–2.74)	1.63 (0.93–2.63)	1.77 (1.04–2.67)	0.35	1.60 (1.01–2.86)	1.70 (0.93–2.59)	1.63 (0.88–2.58)	1.77 (1.04–2.58)	0.36
NFS	−1.46 (−2.89 to −0.43)^†^	−1.48 (−3.40 to −0.32)	−1.69 (−3.22 to −0.29)	−1.58 (−2.30 to −0.86)	0.36	−1.57 (−3.00 to −0.69)	−1.70 (−3.50 to −0.90)	−1.78 (−3.35 to −0.65)	−1.76 (−2.46 to −0.97)	0.66
CAP (dB/m)	312 (267–336)	306 (268–345)^‡^	289 (239–332)	318 (288–347)^‡^	0.140	309 (271–332)	304 (249–333)^‡^	283 (241–320)	312 (292–352)^‡^	0.14
LSM (kPa)	6.4 (5.1–10.2)	6.2 (5.4–8.4)^‡^	6.1 (5.3–11.2)	6.1 (4.0–6.9)^‡^	0.19	5.8 (5.0–7.9)	6.0 (4.6–7.8)^‡^	5.7 (4.9–8.0)	5.9 (4.6–6.9)^‡^	0.35
FAST score	0.39 (0.19–0.62)	0.38 (0.21–0.55)^‡^	0.28 (0.12–0.48)	0.32 (0.12–0.48)^‡^	0.029	0.39 (0.15–0.55)	0.37 (0.16–0.54)^‡^	0.27 (0.12–0.44)	0.33 (0.16–0.50)^‡^	0.064

Data are expressed as the median (IQR).

^†^ Lack of data was seen in one patient.

^‡^ Missing data were found in two patients.

γ‐GTP, γ‐glutamyl transpeptidase; ALBI score, albumin‐bilirubin score; ALP, alkaline phosphatase; ALT, alanine aminotransferase; AST, aspartate aminotransferase; BMI, body mass index; CAP, controlled attenuation parameter; eGFR, estimated glomerular filtration rate; FAST score, FibroScan‐aspartate aminotransferase score; HDL‐C, high‐density lipoprotein cholesterol; IQR, interquartile range; LDL‐C, low‐density lipoprotein cholesterol; LSM, liver stiffness measurement; NFS, non‐alcoholic fatty liver disease fibrosis score; SGLT2, sodium‐glucose co‐transporter‐2; TG, triglyceride.

We excluded the five patients with DM treated with SGLT2 inhibitor and analyzed in remaining 26 patients. The results obtained from these remaining patients were almost in agreement with those obtained from all patients. The value of AST and FAST scores showed a tendency to decrease during pemafibrate treatment (Table 2). The results of post‐hoc analysis were described in Table S1, Supporting information.

### Changes in FAST score, ALBI score, FIB‐4, and NFS at 12 weeks, 24 weeks, and 48 weeks

**Figure 1:**
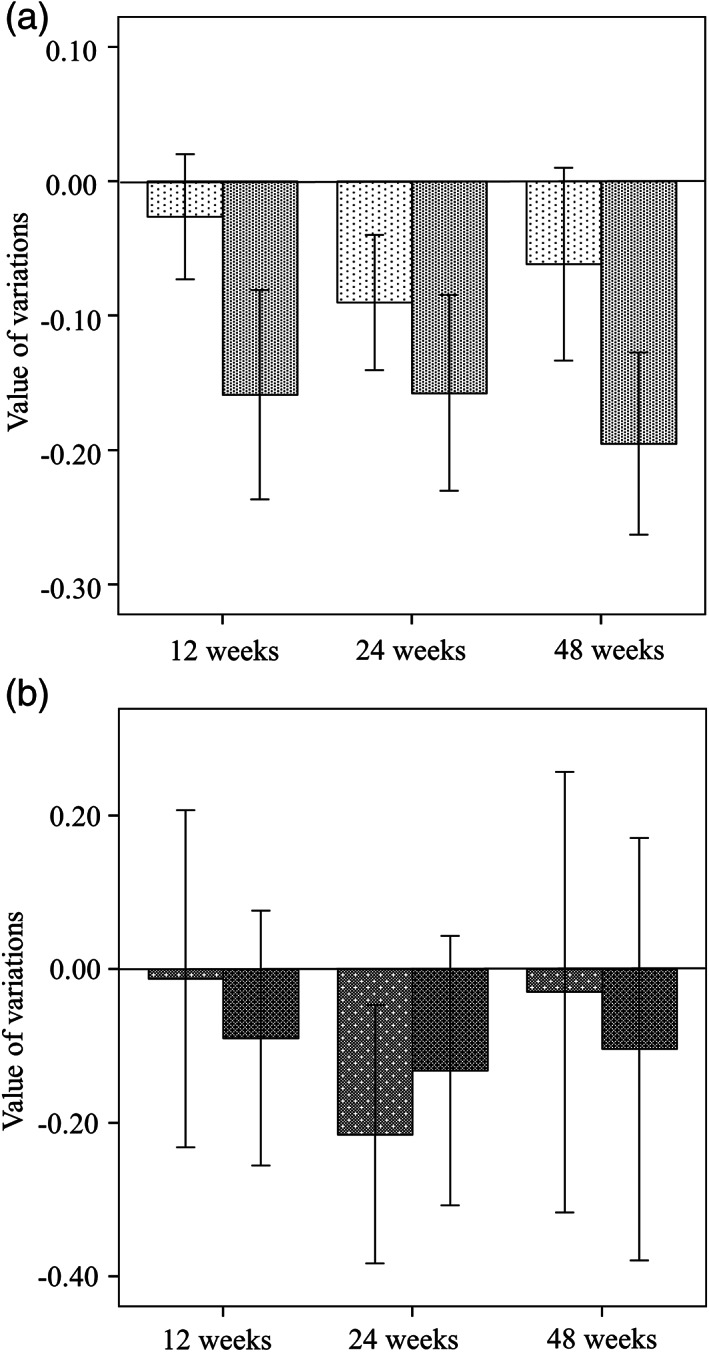
Amounts of changes in each parameter compared to values at pretreatment. FibroScan‐aspartate aminotransferase (FAST) score and albumin‐bilirubin (ALBI) score (a), FIB‐4 and non‐alcoholic fatty liver disease fibrosis score (NFS) (b). a: (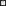), FAST score; (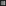), ALBI score. b: (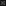), FIB‐4; (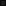), NFS. Bar graphs showed the variations of each parameter and error bars indicated 95% confidence interval. There was lack of data on FAST score at 12 weeks and 48 weeks in two patients.

Figure 1 showed amounts of changes in each parameter compared to values at pretreatment. There was lack of data on FAST score at 12 weeks and 48 weeks in two patients. Mean variations of FAST score were calculated to be −0.03 (95% CI −0.07 to −0.20) at 12 weeks, −0.09 (95% CI −0.14 to −0.04) at 24 weeks, and −0.06 (95% CI −0.13 to 0.01) at 48 weeks. Mean variations of ALBI score was also −0.16 (95% CI −0.23 to −0.08) at 12 weeks, −0.16 (95% CI −0.23 to −0.08) at 24 weeks, and −0.19 (95% CI −0.26 to −0.12) at 48 weeks (Fig. 1a). With respect to liver fibrosis markers, mean variations of FIB‐4 and NFS was −0.01 (95% CI −0.23 to 0.21) and −0.09 (95% CI −0.26 to 0.08) at 12 weeks, and −0.22 (95% CI −0.38 to −0.05) and −0.13 (95% CI −0.31 to 0.04) at 24 weeks, −0.03 (95% CI −0.32 to 0.26) and −0.10 (95% CI −0.38 to 0.17) at 48 weeks, respectively (Fig. 1b).

### Association of delta FAST score and delta ALBI score with variations of other parameters

**Figure 2:**
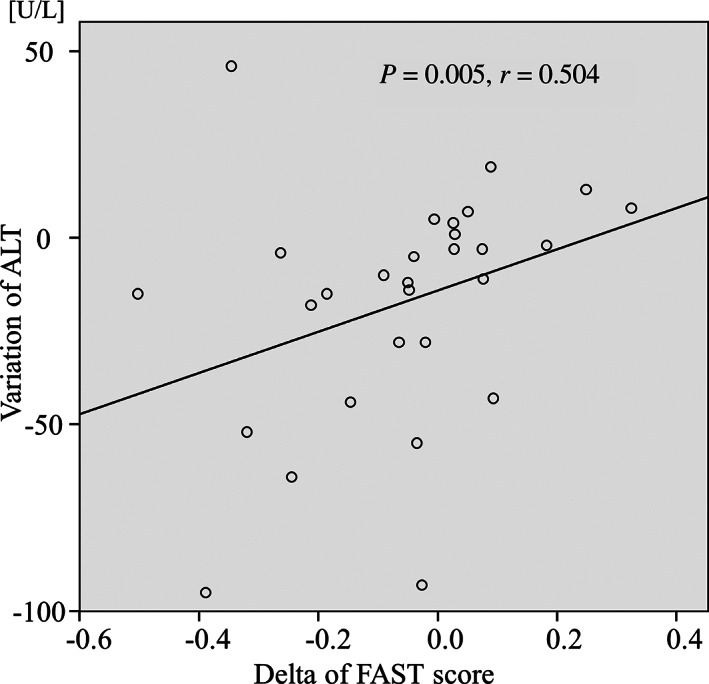
Scatter plots showing the relationships between the variation of the FibroScan‐aspartate aminotransferase (FAST) score and alanine aminotransferase (ALT).

The delta FAST score was found to be correlated with the variations of ALT (*r* = 0.504, *P* = 0.005). There were not any factors associated with the delta ALBI score (Table 3). The scatter plots in Figure 2 show the relationship between the delta FAST score and variation of ALT. At the time of the analysis, no patients developed muscle pain or impairment of the renal function as AEs. Furthermore, there were no AEs leading to dose reduction, interruption, or discontinuation, and all patients received pemafibrate for more than 24 weeks (Table 3).

**Table 3:** Association of the delta FibroScan‐aspartate aminotransferase (FAST) score and delta albumin‐bilirubin (ALBI) score with variation of other parameters

	Delta FAST score		Delta ALBI score	
Variation of parameters	Correlation coefficient	*P*	Correlation coefficient	*P*
Bodyweight	0.162	0.41	0.196	0.31
AST	NA	NA	0.225	0.22
ALT	0.504	0.005	0.149	0.42
ALP	0.150	0.44	0.133	0.47
γ‐GTP	0.248	0.19	0.027	0.88
Platelet	0.040	0.84	0.089	0.64
eGFR	−0.007	0.97	0.157	0.40
TG	0.057	0.77	−0.064	0.73
LDL‐C	−0.004	0.99	−0.286	0.12
HDL‐C	0.013	0.95	−0.317	0.083
Total bilirubin	0.222	0.25	NA	NA
Albumin	−0.080	0.68	NA	NA
ALBI score	0.196	0.31	NA	NA
FIB‐4	NA	NA	0.210	0.26
NFS	−0.034	0.86	−0.021	0.91
LSM	NA	NA	0.104	0.58
CAP	NA	NA	−0.136	0.46
FAST score	NA	NA	−0.043	0.82

γ‐GTP, γ‐glutamyl transpeptidase; ALP, alkaline phosphatase; ALT, alanine aminotransferase; AST, aspartate aminotransferase; CAP, controlled attenuation parameter; eGFR, estimated glomerular filtration rate; HDL‐C, high‐density lipoprotein cholesterol; LDL‐C, low‐density lipoprotein cholesterol; LSM, liver stiffness measurement; NA, not available; NFS, non‐alcoholic fatty liver disease fibrosis score; TG, triglyceride.

## Discussion

The main findings of the current study were that the FAST score was improved during the pemafibrate treatment and was correlated with variation of ALT, which represents the hepatic anti‐inflammatory effect of pemafibrate. While our previous report^15^ revealed the efficacy and safety of pemafibrate for biopsy‐proven NASH patients, whether or not the therapeutic efficacy of pemafibrate could prevent NAFLD progression remains unknown. Accordingly, we expanded the patient cohort and treatment period in the present study and focused on the relationship between the effect of pemafibrate and changes of the FAST score, which effectively stratifies patients with risk of progressive NASH. Because SGLT2 inhibitor, which was used for the treatment of DM, has an effect on transaminase and hepatic fat content,^22^, ^23^ we analyzed the 26 pemafibrate‐treated NAFLD patients who did not receive the SGLT2 inhibitor, resulting in a tendency to improve the FAST score. The reason for the deficient of significance was probably due to the lack of statistical power. We also used the ALBI score, which is a simple assessment of the preserved liver function, to evaluate the changes in the liver function, which demonstrated the improvement due to pemafibrate. To our knowledge, this is the first report to assess the efficacy of pemafibrate therapy in NAFLD patients using the FAST score.

In addition to the effect of reducing TG and increasing HDL‐C, pemafibrate significantly reduced the ALT and γ‐GTP values in comparison to the pretreatment values according to a phase III study.^11^ Investigators^12^, ^13^, ^14^ reported that pemafibrate also improved hepatobiliary enzyme levels without a significant body reduction in NAFLD patients with hypertriglyceridemia, which was in agreement with the results of the present study. Although the clear mechanism through which the hepatobiliary enzyme levels are improved remains unknown, Honda *et al*. reported that pemafibrate improved the pathological findings, including the NAS, hepatic steatosis, and hepatocyte ballooning on a rodent model of NASH.^24^ Sasaki *et al*. showed that pemafibrate improved F4/80‐positive macrophage accumulation and NAS without decreasing TG accumulation in the liver in a mouse model of NASH.^25^ They also showed that pemafibrate increased the number of lipid droplets and decreased the lipid droplet size, resulting in the improvement of macrovesicular steatosis.^25^ Given the findings of these studies associated with a mouse model of NASH, pemafibrate might ameliorate the liver inflammation and reduce NASH with or without reducing hepatic steatosis, achieving the improvement of the liver function in the clinical setting.

Newsome *et al*. proposed the FAST score as a useful tool for non‐invasively identifying NASH patients who are at risk of disease progression.^16^ The FAST score showed good diagnostic performance with an area under the receiver operating curve (AUROC) of 0.80 (95% CI 0.76–0.85).^16^ They also showed that a cutoff value of 0.35 achieved 90% sensitivity, while a cutoff value of 0.67 achieved 90% specificity.^16^ A retrospective study of a Japanese NAFLD cohort^26^ showed that the FAST score provided an AUROC of 0.76, which was considered to be in agreement with the results of a study reported by Newsome *et al*.^16^ Accordingly, the FAST score is a novel index of steatohepatitis that predicts disease progression. In addition, Ogawa *et al*.^27^ analyzed 290 patients with chronic hepatitis C (CH‐C) after they achieved a sustained virological response (SVR), and reported that the cumulative incidence of HCC was significantly higher in patients with a FAST score ≥ 0.35 than those with a FAST score < 0.35 (cumulative rate at 5 years, 27.9 vs. 3.5%, *P* < 0.001). They also revealed that the FAST score was a predictive factor associated with carcinogenesis in a multivariate analysis among CH‐C patients with SVR.^27^ In the current study, the FAST score decreased during pemafibrate treatment and was correlated with the variation of ALT, indicating that the hepatic anti‐inflammatory effect of pemafibrate could prevent NAFLD progression. Further study was warranted to confirm whether the FAST score predicts the development of HCC in NAFLD patients.

The ALBI score, which consists of serum level of albumin and total bilirubin, is a simple assessment of the preserved liver function.^18^ According to previous reports, the serum level of albumin was elevated in patients with NAFLD who were treated with pemafibrate, which might be due to the continuing amelioration of liver inflammation.^12^, ^13^ Shinozaki *et al*.^13^ also reported that the ALBI score was also improved at 3 months and that the delta ALBI score was correlated with delta ALP, which was presumed to be due to the pharmacological effect of pemafibrate. In the current study, the level of albumin and the ALBI score was significantly improved in comparison to the pretreatment values and were maintained at 48 weeks while we failed to find factors correlated with delta ALBI score. Based on the previous studies and the present results, pemafibrate could improve and sustain the preserved liver function as well as liver enzyme levels via the amelioration of liver inflammation.

The present study was associated with some limitations. First, the current study was retrospective in nature and the study population was relatively small. Second, the observation period was relatively short. Third, vitamin E, RAS inhibitor, and statin were concomitantly used in some participated patients. Because these drugs possibly improve the NAFLD diseases based on the previous studies,^28^, ^29^, ^30^ their therapeutic efficacy might affect the present results.

In conclusion, pemafibrate improved the FAST score as well as the liver function due to the hepatic anti‐inflammatory effect, indicating that pemafibrate may prevent disease progression in NAFLD patients with hypertriglyceridemia.

## Supporting information

**Table S1.** Supporting information.
